# An *S*-type singular value inclusion set for rectangular tensors

**DOI:** 10.1186/s13660-017-1421-0

**Published:** 2017-06-17

**Authors:** Caili Sang

**Affiliations:** College of Data Science and Information Engineering, Guizhou Minzu University, Guiyang, Guizhou 550025 P.R. China

**Keywords:** 15A18, 15A69, rectangular tensors, nonnegative tensors, singular value, inclusion set

## Abstract

An *S*-type singular value inclusion set for rectangular tensors is given. Based on the set, new upper and lower bounds for the largest singular value of nonnegative rectangular tensors are obtained and proved to be sharper than some existing results. Numerical examples are given to verify the theoretical results.

## Introduction

Let $\mathbb{R} (\mathbb{C})$ be the real (complex) field, $p,q,m,n$ be positive integers, $l=p+q$, $m,n\geq2$ and $N=\{1,2,\ldots,n\}$. We call $\mathcal{A}=(a_{i_{1}\cdots i_{p}j_{1}\cdots j_{q}})$ a real $(p,q)$th order $m\times n$ dimensional rectangular tensor, or simply a real rectangular tensor, denoted by $\mathcal{A}\in\mathbb{R}^{[p,q;m,n]}$, if

$$a_{i_{1}\cdots i_{p}j_{1}\cdots j_{q}}\in\mathbb{R}, \quad 1\leq i_{1},\ldots, i_{p} \leq m, 1\leq j_{1},\ldots, j_{q}\leq n. $$

When $p=q=1, \mathcal{A}$ is simply a real $m\times n$ rectangular matrix. This justifies the word ‘rectangular’. We call $\mathcal{A}$ nonnegative, denoted by $\mathcal{A}\in\mathbb {R}^{[p,q;m,n]}_{+}$, if each of its entries $a_{i_{1}\cdots i_{p}j_{1}\cdots j_{q}}\geq0$.

For any vectors $x=(x_{1},x_{2},\ldots, x_{m})^{\mathrm{T}}$, $y=(y_{1},y_{2},\ldots , y_{n})^{\mathrm{T}}$ and any real number *α*, denote $x^{[\alpha ]}=(x_{1}^{\alpha},x_{2}^{\alpha},\ldots,x_{m}^{\alpha})^{\mathrm{T}}$ and $y^{[\alpha]}=(y_{1}^{\alpha}, y_{2}^{\alpha},\ldots, y_{n}^{\alpha})^{\mathrm{T}}$. Let $\mathcal{A}x^{p-1}y^{q}$ be a vector in $\mathbb{R}^{m}$ such that

$$\bigl(\mathcal{A}x^{p-1}y^{q}\bigr)_{i}=\sum _{i_{2},\ldots,i_{p}=1}^{m}\sum_{j_{1},\ldots ,j_{q}=1}^{n}a_{ii_{2}\cdots i_{p}j_{1}\cdots j_{q}}x_{i_{2}} \cdots x_{i_{p}}y_{j_{1}}\cdots y_{j_{q}}, $$

where $i=1,\ldots,m$. Similarly, let $\mathcal{A}x^{p}y^{q-1}$ be a vector in $\mathbb{R}^{n}$ such that

$$\bigl(\mathcal{A}x^{p}y^{q-1}\bigr)_{j}=\sum _{i_{1},\ldots,i_{p}=1}^{m}\sum_{j_{2},\ldots ,j_{q}=1}^{n}a_{i_{1}\cdots i_{p}jj_{2}\cdots j_{q}}x_{i_{1}} \cdots x_{i_{p}}y_{j_{2}}\cdots y_{j_{q}}, $$

where $j=1,\ldots,n$. If there are a number $\lambda\in\mathbb{C}$, vectors $x\in\mathbb{C}^{m}\backslash\{ 0\}$, and $y\in\mathbb{C}^{n}\backslash\{0\}$ such that

$$ \textstyle\begin{cases} \mathcal{A}x^{p-1}y^{q}=\lambda x^{[l-1]}, \\ \mathcal{A}x^{p}y^{q-1}=\lambda y^{[l-1]}, \end{cases} $$

then *λ* is called the singular value of $\mathcal{A}$, and $(x,y)$ is a pair of left and right eigenvectors of $\mathcal{A}$, associated with *λ*, respectively. If $\lambda\in\mathbb{R}, x\in\mathbb{R}^{m}$, and $y\in\mathbb{R}^{n}$, then we say that *λ* is an H-singular value of $\mathcal{A}$, and $(x,y)$ is a pair of left and right H-eigenvectors associated with *λ*, respectively. If a singular value is not an H-singular value, we call it an N-singular value of $\mathcal{A}$ [1]. We call

$$\lambda_{0}=\max\bigl\{ \vert \lambda \vert :\lambda \mbox{ is a singular value of }\mathcal {A}\bigr\} $$

the largest singular value [2].

Note here that the definition of singular values for tensors was first proposed by Lim in [3]. When *l* is even, the definition in [1] is the same as in [3]. When *l* is odd, the definition in [1] is slightly different from that in [3], but parallel to the definition of eigenvalues of square matrices [4]; see [1] for details.

When $m=n$, such real rectangular tensors have a sound application background. For example, the elasticity tensor is a tensor with $p=q=2$ and $m=n=2$ or 3; for details, see [1]. Due to the fact that singular values of rectangular tensors have a wide range of practical applications in the strong ellipticity condition problem in solid mechanics [5, 6] and the entanglement problem in quantum physics [7, 8], very recently, it has attracted attention of researchers [9–17]. Chang *et al*. [1] studied some properties of singular values of rectangular tensors, which include the Perron-Frobenius theorem of nonnegative irreducible tensors. Yang *et al*. [2] extended the Perron-Frobenius theorem of nonnegative irreducible tensors to nonnegative tensors, and gave the upper and lower bounds of the largest singular value of nonnegative rectangular tensors.

Our goal in this paper is to propose a singular value inclusion set for rectangular tensors and use the set to obtain new upper and lower bounds for the largest singular value of nonnegative rectangular tensors.

## Main results

In this section, we begin with some notation. Let $\mathcal{A}\in \mathbb{R}^{[p,q;n,n]}$. For $\forall i,j\in N, i\neq j$, denote

$$\begin{aligned} &R_{i}(\mathcal{A})=\sum_{i_{2},\ldots,i_{p},j_{1},\ldots,j_{q}\in N} \vert a_{ii_{2}\cdots i_{p}j_{1}\cdots j_{q}} \vert , \\ &r_{i}^{j}(\mathcal{A})=\sum_{\delta_{ji_{2}\cdots i_{p}j_{1}\cdots j_{q}}=0} \vert a_{ii_{2}\cdots i_{p}j_{1}\cdots j_{q}} \vert =R_{i}(\mathcal{A})- \vert a_{ij\cdots jj\cdots j} \vert , \\ &C_{j}(\mathcal{A})=\sum_{i_{1},\ldots,i_{p},j_{2},\ldots,j_{q}\in N} \vert a_{i_{1}\cdots i_{p}jj_{2}\cdots j_{q}} \vert , \\ &c_{j}^{i}(\mathcal{A})=\sum_{\delta_{i_{1}\cdots i_{p}ij_{2}\cdots j_{q}}=0} \vert a_{i_{1}\cdots i_{p}jj_{2}\cdots j_{q}} \vert =C_{j}(\mathcal{A})- \vert a_{i\cdots iji\cdots i} \vert , \end{aligned}$$

where

$$\delta_{i_{1}\cdots i_{p}j_{1}\cdots j_{q}}= \textstyle\begin{cases}1&\mbox{if }i_{1}=\cdots=i_{p}=j_{1}=\cdots=j_{q},\\ 0&\mbox{otherwise}. \end{cases} $$

### Theorem 1

*Let* $\mathcal{A}\in\mathbb{R}^{[p,q;n,n]}$, *S* *be a nonempty proper subset of* *N*, *S̄* *be the complement of* *S* *in N*. *Then*

$$\sigma(\mathcal{A})\subseteq\Upsilon^{S}(\mathcal{A})= \biggl(\bigcup _{i\in S,j\in\bar{S}} \bigl(\hat{\Upsilon}_{i,j}(\mathcal {A})\cup\tilde{\Upsilon}_{i,j}(\mathcal{A}) \bigr) \biggr) \cup \biggl( \bigcup_{i\in\bar{S},j\in S} \bigl(\hat{\Upsilon}_{i,j}( \mathcal {A})\cup\tilde{\Upsilon}_{i,j}(\mathcal{A}) \bigr) \biggr), $$

*where*

$$\begin{aligned} &\hat{\Upsilon}_{i,j}(\mathcal{A})= \bigl\{ z\in\mathbb{C}: \bigl( \vert z \vert -r_{i}^{j}(\mathcal{A})\bigr) \vert z \vert \leq \vert a_{ij\cdots jj\cdots j} \vert \max\bigl\{ R_{j}( \mathcal{A}),C_{j}(\mathcal{A})\bigr\} \bigr\} , \\ &\tilde{\Upsilon}_{i,j}(\mathcal{A})= \bigl\{ z\in\mathbb{C}: \bigl( \vert z \vert -c_{i}^{j}(\mathcal{A})\bigr) \vert z \vert \leq \vert a_{j\cdots jij\cdots j} \vert \max\bigl\{ R_{j}( \mathcal{A}),C_{j}(\mathcal{A})\bigr\} \bigr\} . \end{aligned}$$

### Proof

For any $\lambda\in\sigma(\mathcal{A})$, let $x=(x_{1},x_{2},\ldots ,x_{n})^{\mathrm{T}}\in\mathbb{C}^{n}\backslash\{0\}$ and $y=(y_{1},y_{2},\ldots ,y_{n})^{\mathrm{T}}\in\mathbb{C}^{n}\backslash\{0\}$ be the associated left and right eigenvectors, that is, 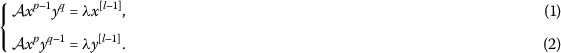 Let

$$\begin{aligned} & \vert x_{s} \vert =\max_{i\in S}\bigl\{ \vert x_{i} \vert \bigr\} ,\qquad \vert x_{t} \vert =\max _{i\in\bar{S}}\bigl\{ \vert x_{i} \vert \bigr\} ,\qquad \vert y_{g} \vert =\max_{i\in S}\bigl\{ \vert y_{i} \vert \bigr\} , \qquad \vert y_{h} \vert =\max _{i\in\bar{S}}\bigl\{ \vert y_{i} \vert \bigr\} , \\ &w_{i}=\max_{i\in N}\bigl\{ \vert x_{i} \vert , \vert y_{i} \vert \bigr\} ,\qquad w_{S}=\max_{i\in S}\{ w_{i}\},\qquad w_{\bar{S}}=\max_{i\in\bar{S}}\{w_{i}\}. \end{aligned}$$

Then, at least one of $\vert x_{s} \vert $ and $\vert x_{t} \vert $ is nonzero, and at least one of $\vert y_{g} \vert $ and $\vert y_{h} \vert $ is nonzero. We divide the proof into four parts.

Case I: Suppose that $w_{S}= \vert x_{s} \vert , w_{\bar{S}}= \vert x_{t} \vert $, then $\vert x_{s} \vert \geq \vert y_{s} \vert , \vert x_{t} \vert \geq \vert y_{t} \vert $.

(i) If $\vert x_{s} \vert \geq \vert x_{t} \vert $, then $\vert x_{s} \vert =\max_{i\in N} \{w_{i}\}$. The *s*th equality in (1) is

$$\lambda x_{s}^{l-1}=\sum_{\delta_{ti_{2}\cdots i_{p}j_{1}\cdots j_{q}}=0}a_{si_{2}\cdots i_{p}j_{1}\cdots j_{q}}x_{i_{2}} \cdots x_{i_{p}}y_{j_{1}}\cdots y_{j_{q}}+a_{st\cdots tt\cdots t}x_{t}^{p-1}y_{t}^{q}. $$

Taking modulus in the above equation and using the triangle inequality give

$$\begin{aligned} \vert \lambda \vert \vert x_{s} \vert ^{l-1}\leq{}& \sum_{\delta_{ti_{2}\cdots i_{p}j_{1}\cdots j_{q}}=0} \vert a_{si_{2}\cdots i_{p}j_{1}\cdots j_{q}} \vert \vert x_{i_{2}} \vert \cdots \vert x_{i_{p}} \vert \vert y_{j_{1}} \vert \cdots y_{j_{q}}| \\ &{}+ \vert a_{st\cdots tt\cdots t} \vert \vert x_{t} \vert ^{p-1} \vert y_{t} \vert ^{q} \\ \leq{}&\sum_{\delta_{ti_{2}\cdots i_{p}j_{1}\cdots j_{q}}=0} \vert a_{si_{2}\cdots i_{p}j_{1}\cdots j_{q}} \vert \vert x_{s} \vert ^{l-1}+ \vert a_{st\cdots tt\cdots t} \vert \vert x_{t} \vert ^{l-1} \\ ={}&r_{s}^{t}(\mathcal{A}) \vert x_{s} \vert ^{l-1}+ \vert a_{st\cdots tt\cdots t} \vert \vert x_{t} \vert ^{l-1}, \end{aligned}$$

i.e.,

3 $$\begin{aligned} \bigl( \vert \lambda \vert -r_{s}^{t}( \mathcal{A})\bigr) \vert x_{s} \vert ^{l-1}\leq \vert a_{st\cdots tt\cdots t} \vert \vert x_{t} \vert ^{l-1}. \end{aligned}$$

If $\vert x_{t} \vert =0$, then $\vert \lambda \vert -r_{s}^{t}(\mathcal{A})\leq0$ as $\vert x_{s} \vert >0$, and it is obvious that

$$\begin{aligned} \bigl( \vert \lambda \vert -r_{s}^{t}(\mathcal{A})\bigr) \vert \lambda \vert \leq0\leq \vert a_{st\cdots tt\cdots t} \vert R_{t}(\mathcal{A}), \end{aligned}$$

which implies that $\lambda\in\hat{\Upsilon}_{s,t}(\mathcal{A})$. Otherwise, $\vert x_{t} \vert >0$. Moreover, from the *t*th equality in (1), we can get

4 $$\begin{aligned} \vert \lambda \vert \vert x_{t} \vert ^{l-1}\leq{}&\sum_{i_{2},\ldots i_{p},j_{1},\ldots, j_{q}\in N} \vert a_{ti_{2}\cdots i_{p}j_{1}\cdots j_{q}} \vert \vert x_{i_{2}} \vert \cdots \vert x_{i_{p}} \vert \vert y_{j_{1}} \vert \cdots \vert y_{j_{q}} \vert \\ \leq{}& R_{t}(\mathcal{A}) \vert x_{s} \vert ^{l-1}. \end{aligned}$$

Multiplying (3) by (4) and noting that $\vert x_{s} \vert ^{l-1} \vert x_{t} \vert ^{l-1}>0$, we have

$$\begin{aligned} \bigl( \vert \lambda \vert -r_{s}^{t}(\mathcal{A})\bigr) \vert \lambda \vert \leq \vert a_{st\cdots tt\cdots t} \vert R_{t}( \mathcal{A}), \end{aligned}$$

which also implies that $\lambda\in\hat{\Upsilon}_{s,t}(\mathcal{A})\subseteq\bigcup_{i\in S,j\in\bar{S}}\hat{\Upsilon}_{i,j}(\mathcal{A})$.

(ii) If $\vert x_{t} \vert \geq \vert x_{s} \vert $, then $\vert x_{t} \vert =\max_{i\in N} \{w_{i}\}$. Similarly, we can get

$$\begin{aligned} \bigl( \vert \lambda \vert -r_{t}^{s}(\mathcal{A})\bigr) \vert \lambda \vert \leq \vert a_{ts\cdots ss\cdots s} \vert R_{s}( \mathcal{A}), \end{aligned}$$

and $\lambda\in\hat{\Upsilon}_{t,s}(\mathcal{A})\subseteq\bigcup_{i\in \bar{S},j\in S}\hat{\Upsilon}_{i,j}(\mathcal{A})$.

Case II: Suppose that $w_{S}= \vert y_{g} \vert , w_{\bar{S}}= \vert y_{h} \vert $, then $\vert y_{g} \vert \geq \vert x_{g} \vert , \vert y_{h} \vert \geq \vert x_{h} \vert $.

(i) If $\vert y_{g} \vert \geq \vert y_{h} \vert $, then $\vert y_{g} \vert =\max_{i\in N} \{w_{i}\}$. The *g*th equality in (2) is

$$\lambda y_{g}^{l-1}=\sum_{\delta_{i_{1}\cdots i_{p}hj_{2}\cdots j_{q}}=0}a_{i_{1}\cdots i_{p}gj_{2}\cdots j_{q}}x_{i_{1}} \cdots x_{i_{p}}y_{j_{2}}\cdots y_{j_{q}}+a_{h\cdots hgh\cdots h}x_{h}^{p}y_{h}^{q-1}. $$

Taking modulus in the above equation and using the triangle inequality give

$$\begin{aligned} \vert \lambda \vert \vert y_{g} \vert ^{l-1}\leq{}& \sum_{\delta_{i_{1}\cdots i_{p}hj_{2}\cdots j_{q}}=0} \vert a_{i_{1}\cdots i_{p}gj_{2}\cdots j_{q}} \vert \vert x_{i_{1}} \vert \cdots \vert x_{i_{p}} \vert \vert y_{j_{2}} \vert \cdots \vert y_{j_{q}} \vert \\ &{}+ \vert a_{h\cdots hgh\cdots h} \vert \vert x_{h} \vert ^{p} \vert y_{h} \vert ^{q-1} \\ \leq{}&\sum_{\delta_{i_{1}\cdots i_{p}hj_{2}\cdots j_{q}}=0} \vert a_{i_{1}\cdots i_{p}gj_{2}\cdots j_{q}} \vert \vert y_{g} \vert ^{l-1}+ \vert a_{h\cdots hgh\cdots h} \vert \vert y_{h} \vert ^{l-1} \\ ={}&c_{g}^{h}(\mathcal{A}) \vert y_{g} \vert ^{l-1}+ \vert a_{h\cdots hgh\cdots h} \vert \vert y_{h} \vert ^{l-1}, \end{aligned}$$

i.e.,

5 $$\begin{aligned} \bigl( \vert \lambda \vert -c_{g}^{h}( \mathcal{A})\bigr) \vert y_{g} \vert ^{l-1}\leq \vert a_{h\cdots hgh\cdots h} \vert \vert y_{h} \vert ^{l-1}. \end{aligned}$$

If $\vert y_{h} \vert =0$, then $\vert \lambda \vert -c_{g}^{h}(\mathcal{A})\leq0$ as $\vert y_{g} \vert >0$, and furthermore

$$\begin{aligned} \bigl( \vert \lambda \vert -c_{g}^{h}(\mathcal{A}) \bigr) \vert \lambda \vert \leq0\leq \vert a_{h\cdots hgh\cdots h} \vert C_{h}(\mathcal{A}), \end{aligned}$$

which implies that $\lambda\in\tilde{\Upsilon}_{g,h}(\mathcal{A})$. Otherwise, $\vert y_{h} \vert >0$. Moreover, from the *h*th equality in (2), we can get

6 $$\begin{aligned} \vert \lambda \vert \vert y_{h} \vert ^{l-1}\leq{}&\sum_{i_{1},\ldots, i_{p},j_{2},\ldots, j_{q}\in N} \vert a_{i_{1}\cdots i_{p}hj_{2}\cdots j_{q}} \vert \vert x_{i_{1}} \vert \cdots \vert x_{i_{p}} \vert \vert y_{j_{2}} \vert \cdots \vert y_{j_{q}} \vert \\ \leq{}& C_{h}(\mathcal{A}) \vert y_{g} \vert ^{l-1}. \end{aligned}$$

Multiplying (5) by (6) and noting that $\vert y_{g} \vert ^{l-1} \vert y_{h} \vert ^{l-1}>0$, we have

$$\begin{aligned} \bigl( \vert \lambda \vert -c_{g}^{h}(\mathcal{A}) \bigr) \vert \lambda \vert \leq \vert a_{h\cdots hgh\cdots h} \vert C_{h}(\mathcal{A}), \end{aligned}$$

which also implies that $\lambda\in\tilde{\Upsilon}_{g,h}(\mathcal {A})\subseteq\bigcup_{i\in S,j\in\bar{S}}\tilde{\Upsilon }_{i,j}(\mathcal{A})$.

(ii) If $\vert y_{h} \vert \geq \vert y_{g} \vert $, then $\vert y_{h} \vert =\max_{i\in N} \{w_{i}\}$. Similarly, we can get

$$\begin{aligned} \bigl( \vert \lambda \vert -c_{h}^{g}(\mathcal{A}) \bigr) \vert \lambda \vert \leq \vert a_{g\cdots ghg\cdots g} \vert C_{g}(\mathcal{A}), \end{aligned}$$

and $\lambda\in\tilde{\Upsilon}_{h,g}(\mathcal{A})\subseteq\bigcup_{i\in \bar{S},j\in S}\tilde{\Upsilon}_{i,j}(\mathcal{A})$.

Case III: Suppose that $w_{S}= \vert x_{s} \vert , w_{\bar{S}}= \vert y_{h} \vert $, then $\vert x_{s} \vert \geq \vert y_{s} \vert , \vert y_{h} \vert \geq \vert x_{h} \vert $. If $\vert x_{s} \vert \geq \vert y_{h} \vert $, then $\vert x_{s} \vert =\max_{i\in N} \{w_{i}\}$. Similar to the proof of (3) and (6), we have

$$\begin{aligned} \bigl( \vert \lambda \vert -r_{s}^{h}(\mathcal{A}) \bigr) \vert x_{s} \vert ^{l-1}\leq \vert a_{sh\cdots hh\cdots h} \vert \vert y_{h} \vert ^{l-1} \end{aligned}$$

and

$$\begin{aligned} \vert \lambda \vert \vert y_{h} \vert ^{l-1}\leq C_{h}(\mathcal{A}) \vert x_{s} \vert ^{l-1}. \end{aligned}$$

Furthermore, we have

$$\begin{aligned} \bigl( \vert \lambda \vert -r_{s}^{h}(\mathcal{A}) \bigr) \vert \lambda \vert \leq \vert a_{sh\cdots hh\cdots h} \vert C_{h}(\mathcal{A}), \end{aligned}$$

which implies that $\lambda\in\hat{\Upsilon}_{s,h}(\mathcal{A})\subseteq\bigcup_{i\in S,j\in\bar{S}}\hat{\Upsilon}_{i,j}(\mathcal{A})$. And if $\vert y_{h} \vert \geq \vert x_{s} \vert $, then $\vert y_{h} \vert =\max_{i\in N} \{w_{i}\}$. Similarly, we can get

$$\begin{aligned} \bigl( \vert \lambda \vert -c_{h}^{s}(\mathcal{A}) \bigr) \vert \lambda \vert \leq \vert a_{s\cdots shs\cdots s} \vert R_{s}(\mathcal{A}), \end{aligned}$$

which implies that $\lambda\in\tilde{\Upsilon}_{h,s}(\mathcal{A})\subseteq\bigcup_{i\in \bar{S},j\in S}\tilde{\Upsilon}_{i,j}(\mathcal{A})$.

Case IV: Suppose that $w_{S}= \vert y_{g} \vert , w_{\bar{S}}= \vert x_{t} \vert $, then $\vert y_{g} \vert \geq \vert x_{g} \vert , \vert x_{t} \vert \geq \vert y_{t} \vert $. If $\vert y_{g} \vert \geq \vert x_{t} \vert $, then $\vert y_{g} \vert =\max_{i\in N} \{w_{i}\}$. Similar to the proof of (5) and (4), we have

$$\begin{aligned} \bigl( \vert \lambda \vert -c_{g}^{t}(\mathcal{A}) \bigr) \vert y_{g} \vert ^{l-1}\leq \vert a_{t\cdots tgt\cdots t} \vert \vert x_{t} \vert ^{l-1} \end{aligned}$$

and

$$\begin{aligned} \vert \lambda \vert \vert x_{t} \vert ^{l-1}\leq R_{t}(\mathcal{A}) \vert y_{g} \vert ^{l-1}. \end{aligned}$$

Furthermore, we have

$$\begin{aligned} \bigl( \vert \lambda \vert -c_{g}^{t}(\mathcal{A}) \bigr) \vert \lambda \vert \leq \vert a_{t\cdots tgt\cdots t} \vert R_{t}(\mathcal{A}), \end{aligned}$$

which implies that $\lambda\in\tilde{\Upsilon}_{g,t}(\mathcal {A})\subseteq\bigcup_{i\in\bar{S},j\in S}\tilde{\Upsilon }_{i,j}(\mathcal{A})$. And if $\vert x_{t} \vert \geq \vert y_{g} \vert $, then $\vert x_{t} \vert =\max_{i\in N} \{w_{i}\}$. Similarly, we can get

$$\begin{aligned} \bigl( \vert \lambda \vert -r_{t}^{g}(\mathcal{A}) \bigr) \vert \lambda \vert \leq \vert a_{tg\cdots gg\cdots g} \vert C_{g}(\mathcal{A}), \end{aligned}$$

which implies that $\lambda\in\hat{\Upsilon}_{t,g}(\mathcal {A})\subseteq\bigcup_{i\in\bar{S},j\in S}\hat{\Upsilon}_{i,j}(\mathcal{A})$. The proof is completed. □

Based on Theorem 1, bounds for the largest singular value of nonnegative rectangular tensors are given.

### Theorem 2

*Let* $\mathcal{A}=(a_{i_{1}\cdots i_{m}})\in\mathbb {R}^{[p,q;n,n]}_{+}$, *S* *be a nonempty proper subset of* *N*, *S̄* *be the complement of* *S* *in N*. *Then*

7 $$\begin{aligned} L^{S}(\mathcal{A})\leq\lambda_{0}\leq U^{S}(\mathcal{A}), \end{aligned}$$

*where*

$$\begin{aligned} &L^{S}(\mathcal{A})=\min\bigl\{ \hat{L}^{S}(\mathcal{A}), \hat{L}^{\bar{S}}(\mathcal {A}),\tilde{L}^{S}(\mathcal{A}), \tilde{L}^{\bar{S}}(\mathcal{A})\bigr\} , \\ &U^{S}(\mathcal{A})=\max\bigl\{ \hat{U}^{S}(\mathcal{A}), \hat{U}^{\bar{S}}(\mathcal {A}),\tilde{U}^{S}(\mathcal{A}), \tilde{U}^{\bar{S}}(\mathcal{A})\bigr\} \end{aligned}$$

*and*

$$\begin{aligned} &\hat{L}^{S}(\mathcal{A})=\min_{i\in S,j\in\bar{S}} \frac{1}{2} \bigl\{ r_{i}^{j}(\mathcal{A}) +\bigl[ \bigl(r_{i}^{j}(\mathcal{A})\bigr)^{2}+4a_{ij\cdots jj\cdots j} \min\bigl\{ R_{j}(\mathcal {A}),C_{j}(\mathcal{A})\bigr\} \bigr]^{\frac{1}{2}} \bigr\} , \\ &\tilde{L}^{S}(\mathcal{A})=\min_{i\in S,j\in\bar{S}} \frac{1}{2} \bigl\{ c_{i}^{j}(\mathcal{A}) +\bigl[ \bigl(c_{i}^{j}(\mathcal{A})\bigr)^{2}+4a_{j\cdots jij\cdots j} \min\bigl\{ R_{j}(\mathcal {A}),C_{j}(\mathcal{A})\bigr\} \bigr]^{\frac{1}{2}} \bigr\} , \\ &\hat{U}^{S}(\mathcal{A})=\max_{i\in S,j\in\bar{S}} \frac{1}{2} \bigl\{ r_{i}^{j}(\mathcal{A}) +\bigl[ \bigl(r_{i}^{j}(\mathcal{A})\bigr)^{2}+4a_{ij\cdots jj\cdots j} \max\bigl\{ R_{j}(\mathcal {A}),C_{j}(\mathcal{A})\bigr\} \bigr]^{\frac{1}{2}} \bigr\} , \\ &\tilde{U}^{S}(\mathcal{A})=\max_{i\in S,j\in\bar{S}} \frac{1}{2} \bigl\{ c_{i}^{j}(\mathcal{A}) +\bigl[ \bigl(c_{i}^{j}(\mathcal{A})\bigr)^{2}+4a_{j\cdots jij\cdots j} \max\bigl\{ R_{j}(\mathcal {A}),C_{j}(\mathcal{A})\bigr\} \bigr]^{\frac{1}{2}} \bigr\} . \end{aligned}$$

### Proof

First, we prove that the second inequality in (7) holds. By Theorem 2 in [2], we know that $\lambda_{0}$ is a singular value of $\mathcal{A}$. Hence, by Theorem 1, $\lambda_{0}\in\Upsilon^{S}(\mathcal{A})$, that is,

$$\begin{aligned} &\lambda_{0}\in\bigcup_{i\in S,j\in\bar{S}} \bigl(\hat{ \Upsilon }_{i,j}(\mathcal{A})\cup\tilde{\Upsilon}_{i,j}( \mathcal{A}) \bigr) \quad\mbox{or}\\ & \lambda_{0}\in\bigcup _{i\in\bar{S},j\in S} \bigl(\hat{\Upsilon }_{i,j}(\mathcal{A})\cup \tilde{\Upsilon}_{i,j}(\mathcal{A}) \bigr). \end{aligned}$$

If $\lambda_{0}\in\bigcup_{i\in S,j\in\bar{S}} (\hat{\Upsilon }_{i,j}(\mathcal{A})\cup\tilde{\Upsilon}_{i,j}(\mathcal{A}) )$, then there are $i\in S,j\in\bar{S}$ such that $\lambda_{0}\in\hat{\Upsilon}_{i,j}(\mathcal{A})$ or $\lambda_{0}\in\tilde {\Upsilon}_{i,j}(\mathcal{A})$. When $\lambda_{0}\in\hat{\Upsilon}_{i,j}(\mathcal{A})$, i.e., $(\lambda_{0}-r_{i}^{j}(\mathcal{A}))\lambda_{0}\leq a_{ij\cdots jj\cdots j}\max \{R_{j}(\mathcal{A}),C_{j}(\mathcal{A})\}$, then solving $\lambda_{0}$ gives

$$\begin{aligned} \lambda_{0}&\leq \frac{1}{2} \bigl\{ r_{i}^{j}( \mathcal{A}) +\bigl[\bigl(r_{i}^{j}(\mathcal{A}) \bigr)^{2}+4a_{ij\cdots jj\cdots j}\max\bigl\{ R_{j}(\mathcal {A}),C_{j}(\mathcal{A})\bigr\} \bigr]^{\frac{1}{2}} \bigr\} \\ &\leq \max_{i\in S,j\in\bar{S}}\frac{1}{2} \bigl\{ r_{i}^{j}( \mathcal{A}) +\bigl[\bigl(r_{i}^{j}(\mathcal{A}) \bigr)^{2}+4a_{ij\cdots jj\cdots j}\max\bigl\{ R_{j}(\mathcal {A}),C_{j}(\mathcal{A})\bigr\} \bigr]^{\frac{1}{2}} \bigr\} \\ &=\hat{U}^{S}(\mathcal{A}). \end{aligned}$$

When $\lambda_{0}\in\tilde{\Upsilon}_{i,j}(\mathcal{A})$, i.e., $(\lambda_{0}-c_{i}^{j}(\mathcal{A}))\lambda_{0}\leq a_{j\cdots jij\cdots j}\max \{R_{j}(\mathcal{A}),C_{j}(\mathcal{A})\}$, then solving $\lambda_{0}$ gives

$$\begin{aligned} \lambda_{0}&\leq \frac{1}{2} \bigl\{ c_{i}^{j}( \mathcal{A}) +\bigl[\bigl(c_{i}^{j}(\mathcal{A}) \bigr)^{2}+4a_{j\cdots jij\cdots j}\max\bigl\{ R_{j}(\mathcal {A}),C_{j}(\mathcal{A})\bigr\} \bigr]^{\frac{1}{2}} \bigr\} \\ &\leq \max_{i\in S,j\in\bar{S}}\frac{1}{2} \bigl\{ c_{i}^{j}( \mathcal{A}) +\bigl[\bigl(c_{i}^{j}(\mathcal{A}) \bigr)^{2}+4a_{j\cdots jij\cdots j}\max\bigl\{ R_{j}(\mathcal {A}),C_{j}(\mathcal{A})\bigr\} \bigr]^{\frac{1}{2}} \bigr\} \\ &=\tilde{U}^{S}(\mathcal{A}). \end{aligned}$$

And if $\lambda_{0}\in\bigcup_{i\in\bar{S},j\in S} (\hat{\Upsilon }_{i,j}(\mathcal{A})\cup\tilde{\Upsilon}_{i,j}(\mathcal{A}) )$, similarly, we can obtain that $\lambda_{0}\leq\hat{U}^{\bar{S}}(\mathcal{A})$ and $\lambda_{0}\leq\tilde {U}^{\bar{S}}(\mathcal{A})$.

Second, we prove that the first inequality in (7) holds. Assume that $\mathcal{A}$ is an irreducible nonnegative rectangular tensor, by Theorem 6 of [1], then $\lambda_{0}>0$ with two positive left and right associated eigenvectors $x=(x_{1},x_{2},\ldots,x_{n})^{\mathrm{T}}$ and $y=(y_{1},y_{2},\ldots,y_{n})^{\mathrm{T}}$. Let

$$\begin{aligned} &x_{s}=\min_{i\in S}\{x_{i}\},\qquad x_{t}=\min_{i\in\bar{S}}\{ x_{i}\},\qquad y_{g}=\min_{i\in S}\{y_{i}\},\qquad y_{h}=\min_{i\in\bar{S}}\{y_{i}\}, \\ & w_{i}=\min_{i\in N}\{x_{i},y_{i} \} ,\qquad w_{S}=\min_{i\in S}\{w_{i}\},\qquad w_{\bar{S}}=\min_{i\in\bar{S}}\{w_{i}\}. \end{aligned}$$

We divide the proof into four parts.

Case I: Suppose that $w_{S}=x_{s}, w_{\bar{S}}=x_{t}$, then $y_{s}\geq x_{s}, y_{t}\geq x_{t}$.

(i) If $x_{t}\geq x_{s}$, then ${x_{s}}=\min_{i\in N} \{w_{i}\}$. From the *s*th equality in (1), we have

$$\begin{aligned} \lambda_{0} x_{s}^{l-1}&=\sum _{\delta_{ti_{2}\cdots i_{p}j_{1}\cdots j_{q}}=0}a_{si_{2}\cdots i_{p}j_{1}\cdots j_{q}}x_{i_{2}}\cdots x_{i_{p}}y_{j_{1}}\cdots y_{j_{q}} +a_{st\cdots tt\cdots t}x_{t}^{p-1}y_{t}^{q} \\ &\geq \sum_{\delta_{ti_{2}\cdots i_{p}j_{1}\cdots j_{q}}=0}a_{si_{2}\cdots i_{p}j_{1}\cdots j_{q}}x_{s}^{l-1}+a_{st\cdots tt\cdots t}x_{t}^{l-1} \\ &= r_{s}^{t}(\mathcal{A})x_{s}^{l-1}+a_{st\cdots tt\cdots t}x_{t}^{l-1}, \end{aligned}$$

i.e.,

8 $$\begin{aligned} \bigl(\lambda_{0}-r_{s}^{t}( \mathcal{A})\bigr)x_{s}^{l-1}\geq a_{st\cdots tt\cdots t}x_{t}^{l-1}. \end{aligned}$$

Moreover, from the *t*th equality in (1), we can get

9 $$\begin{aligned} \lambda_{0} x_{t}^{l-1}=\sum _{i_{2},\ldots i_{p},j_{1},\ldots, j_{q}\in N}a_{ti_{2}\cdots i_{p}j_{1}\cdots j_{q}}x_{i_{2}}\cdots x_{i_{p}}y_{j_{1}}\cdots y_{j_{q}}\geq R_{t}( \mathcal{A})x_{s}^{l-1}. \end{aligned}$$

Multiplying (8) by (9) and noting that $x_{s}^{l-1}x_{t}^{l-1}>0$, we have

$$\begin{aligned} \bigl(\lambda_{0}-r_{s}^{t}(\mathcal{A})\bigr) \lambda_{0}\geq a_{st\cdots tt\cdots t}R_{t}(\mathcal{A}). \end{aligned}$$

Then solving for $\lambda_{0}$ gives

$$\begin{aligned} \lambda_{0}(\mathcal{A})&\geq \frac{1}{2} \bigl\{ r_{s}^{t}(\mathcal{A}) +\bigl[\bigl(r_{s}^{t}( \mathcal{A})\bigr)^{2}+4a_{st\cdots tt\cdots t}R_{t}(\mathcal {A}) \bigr]^{\frac{1}{2}} \bigr\} \\ &\geq \min_{i\in S,j\in\bar{S}}\frac{1}{2} \bigl\{ r_{i}^{j}( \mathcal{A}) +\bigl[\bigl(r_{i}^{j}(\mathcal{A}) \bigr)^{2}+4a_{ij\cdots jj\cdots j}R_{j}(\mathcal {A}) \bigr]^{\frac{1}{2}} \bigr\} \geq \hat{L}^{S}(\mathcal{A}). \end{aligned}$$

(ii) If $x_{s}\geq x_{t}$, then $x_{t}=\min_{i\in N} \{w_{i}\}$. Similarly, we can get

$$\begin{aligned} \lambda_{0}(\mathcal{A})&\geq \frac{1}{2} \bigl\{ r_{t}^{s}(\mathcal{A}) +\bigl[\bigl(r_{t}^{s}( \mathcal{A})\bigr)^{2}+4a_{ts\cdots ss\cdots s}R_{s}(\mathcal {A}) \bigr]^{\frac{1}{2}} \bigr\} \\ &\geq \min_{i\in\bar{S},j\in S}\frac{1}{2} \bigl\{ r_{i}^{j}( \mathcal{A}) +\bigl[\bigl(r_{i}^{j}(\mathcal{A}) \bigr)^{2}+4a_{ij\cdots jj\cdots j}R_{j}(\mathcal {A}) \bigr]^{\frac{1}{2}} \bigr\} \geq \hat{L}^{\bar{S}}(\mathcal{A}). \end{aligned}$$

Case II: Suppose that $w_{S}=y_{g}, w_{\bar{S}}=y_{h}$, then $x_{g}\geq y_{g}, x_{h}\geq y_{h}$.

(i) If $y_{h}\geq y_{g}$, then $y_{g}=\min_{i\in N} \{w_{i}\}$. From the *g*th equality in (2), we have

$$\begin{aligned} \lambda_{0} y_{g}^{l-1} =&\sum _{\delta_{i_{1}\cdots i_{p}hj_{2}\cdots j_{q}}=0}a_{i_{1}\cdots i_{p}gj_{2}\cdots j_{q}}x_{i_{1}}\cdots x_{i_{p}}y_{j_{2}}\cdots y_{j_{q}}+a_{h\cdots hgh\cdots h}x_{h}^{p}y_{h}^{q-1} \\ \geq&\sum_{\delta_{i_{1}\cdots i_{p}hj_{2}\cdots j_{q}}=0}a_{i_{1}\cdots i_{p}gj_{2}\cdots j_{q}}y_{g}^{l-1}+a_{h\cdots hgh\cdots h}y_{h}^{l-1} \\ =&c_{g}^{h}(\mathcal{A})y_{g}^{l-1}+a_{h\cdots hgh\cdots h}y_{h}^{l-1}, \end{aligned}$$

i.e.,

10 $$\begin{aligned} \bigl(\lambda_{0}-c_{g}^{h}( \mathcal{A})\bigr)y_{g}^{l-1}\geq a_{h\cdots hgh\cdots h}y_{h}^{l-1}. \end{aligned}$$

Moreover, from the *h*th equality in (2), we can get

11 $$\begin{aligned} \lambda_{0}y_{h}^{l-1}=\sum _{i_{1},\ldots, i_{p},j_{2},\ldots, j_{q}\in N}a_{i_{1}\cdots i_{p}hj_{2}\cdots j_{q}}x_{i_{1}}\cdots x_{i_{p}}y_{j_{2}}\cdots y_{j_{q}}\geq C_{h}( \mathcal{A})y_{g}^{l-1}. \end{aligned}$$

Multiplying (10) by (11) and noting that $y_{g}^{l-1}y_{h}^{l-1}>0$, we have

$$\begin{aligned} \bigl(\lambda_{0}-c_{g}^{h}(\mathcal{A})\bigr) \lambda_{0}\geq a_{h\cdots hgh\cdots h}C_{h}(\mathcal{A}), \end{aligned}$$

which gives

$$\begin{aligned} \lambda_{0}&\geq \frac{1}{2} \bigl\{ c_{g}^{h}( \mathcal{A}) +\bigl[\bigl(c_{g}^{h}(\mathcal{A}) \bigr)^{2}+4a_{h\cdots hgh\cdots h}C_{h}(\mathcal {A}) \bigr]^{\frac{1}{2}} \bigr\} \\ &\geq \min_{i\in S, j\in\bar{S}}\frac{1}{2} \bigl\{ c_{i}^{j}( \mathcal{A}) +\bigl[\bigl(c_{i}^{j}(\mathcal{A}) \bigr)^{2}+4a_{j\cdots jij\cdots j}C_{j}(\mathcal {A}) \bigr]^{\frac{1}{2}} \bigr\} \\ &\geq \tilde{L}^{S}(\mathcal{A}). \end{aligned}$$

(ii) If $y_{g}\geq y_{h}$, then $y_{h}=\min_{i\in N} \{w_{i}\}$. Similarly, we can get

$$\begin{aligned} \lambda_{0}&\geq \frac{1}{2} \bigl\{ c_{h}^{g}( \mathcal{A}) +\bigl[\bigl(c_{h}^{g}(\mathcal{A}) \bigr)^{2}+4a_{g\cdots ghg\cdots g}C_{g}(\mathcal {A}) \bigr]^{\frac{1}{2}} \bigr\} \\ &\geq \min_{i\in\bar{S}, j\in S}\frac{1}{2} \bigl\{ c_{i}^{j}( \mathcal{A}) +\bigl[\bigl(c_{i}^{j}(\mathcal{A}) \bigr)^{2}+4a_{j\cdots jij\cdots j}C_{j}(\mathcal {A}) \bigr]^{\frac{1}{2}} \bigr\} \\ &\geq \tilde{L}^{\bar{S}}(\mathcal{A}). \end{aligned}$$

Case III: Suppose that $w_{S}=x_{s}, w_{\bar{S}}=y_{h}$, then $y_{s}\geq x_{s}, x_{h}\geq y_{h}$. If $y_{h}\geq x_{s}$, then $x_{s}=\min_{i\in N} \{w_{i}\}$. Similar to the proof of (8) and (11), we have

$$\begin{aligned} \bigl(\lambda_{0}-r_{s}^{h}(\mathcal{A}) \bigr)x_{s}^{l-1}\geq a_{sh\cdots hh\cdots h}y_{h}^{l-1} \end{aligned}$$

and

$$\begin{aligned} \lambda_{0} y_{h}^{l-1}\geq C_{h}( \mathcal{A})x_{s}^{l-1}. \end{aligned}$$

Furthermore, we have

$$\begin{aligned} \bigl(\lambda_{0}-r_{s}^{h}(\mathcal{A})\bigr) \lambda_{0}\geq a_{sh\cdots hh\cdots h}C_{h}(\mathcal{A}) \end{aligned}$$

and

$$\begin{aligned} \lambda_{0}&\geq \frac{1}{2} \bigl\{ r_{s}^{h}( \mathcal{A}) +\bigl[\bigl(r_{s}^{h}(\mathcal{A}) \bigr)^{2}+4a_{sh\cdots hh\cdots h}C_{h}(\mathcal {A}) \bigr]^{\frac{1}{2}} \bigr\} \\ &\geq \min_{i\in S,j\in\bar{S}}\frac{1}{2} \bigl\{ r_{i}^{j}( \mathcal{A}) +\bigl[\bigl(r_{i}^{j}(\mathcal{A}) \bigr)^{2}+4a_{ij\cdots jj\cdots j}C_{j}(\mathcal {A}) \bigr]^{\frac{1}{2}} \bigr\} \\ &\geq \hat{L}^{S}(\mathcal{A}). \end{aligned}$$

And if $x_{s}\geq y_{h}$, then $y_{h}=\min_{i\in N} \{w_{i}\}$. Similarly, we have

$$\begin{aligned} \lambda_{0}&\geq \frac{1}{2} \bigl\{ c_{h}^{s}( \mathcal{A}) +\bigl[\bigl(c_{h}^{s}(\mathcal{A}) \bigr)^{2}+4a_{s\cdots shs\cdots s}R_{s}(\mathcal {A}) \bigr]^{\frac{1}{2}} \bigr\} \\ &\geq \min_{i\in\bar{S},j\in S}\frac{1}{2} \bigl\{ c_{i}^{j}( \mathcal{A}) +\bigl[\bigl(c_{i}^{j}(\mathcal{A}) \bigr)^{2}+4a_{j\cdots jij\cdots j}R_{j}(\mathcal {A}) \bigr]^{\frac{1}{2}} \bigr\} \\ &\geq \tilde{L}^{\bar{S}}(\mathcal{A}). \end{aligned}$$

Case IV: Suppose that $w_{S}=y_{g}, w_{\bar{S}}=x_{t}$, then $x_{g}\geq y_{g}, y_{t}\geq x_{t}$. If $x_{t}\geq y_{g}$, then $y_{g}=\min_{i\in N} \{w_{i}\}$. Similar to the proof of (10) and (9), we have

$$\begin{aligned} \bigl(\lambda_{0}-c_{g}^{t}(\mathcal{A}) \bigr)y_{g}^{l-1}\geq a_{t\cdots tgt\cdots t}x_{t}^{l-1} \end{aligned}$$

and

$$\begin{aligned} \lambda_{0}x_{t}^{l-1}\geq R_{t}( \mathcal{A})y_{g}^{l-1}. \end{aligned}$$

Furthermore, we have

$$\begin{aligned} \bigl(\lambda_{0}-c_{g}^{t}(\mathcal{A})\bigr) \lambda_{0}\geq a_{t\cdots tgt\cdots t}R_{t}(\mathcal{A}) \end{aligned}$$

and

$$\begin{aligned} \lambda_{0}&\geq \frac{1}{2} \bigl\{ c_{g}^{t}( \mathcal{A}) +\bigl[\bigl(c_{g}^{t}(\mathcal{A}) \bigr)^{2}+4a_{t\cdots tgt\cdots t}R_{t}(\mathcal {A}) \bigr]^{\frac{1}{2}} \bigr\} \\ &\geq \min_{i\in S,j\in\bar{S}}\frac{1}{2} \bigl\{ c_{i}^{j}( \mathcal{A}) +\bigl[\bigl(c_{i}^{j}(\mathcal{A}) \bigr)^{2}+4a_{j\cdots jij\cdots t}R_{j}(\mathcal {A}) \bigr]^{\frac{1}{2}} \bigr\} \geq \tilde{L}^{S}(\mathcal{A}). \end{aligned}$$

And if $y_{g}\geq x_{t}$, then $x_{t}=\min_{i\in N} \{w_{i}\}$. Similarly, we have

$$\begin{aligned} \lambda_{0}&\geq \frac{1}{2} \bigl\{ r_{t}^{g}( \mathcal{A}) +\bigl[\bigl(r_{t}^{g}(\mathcal{A}) \bigr)^{2}+4a_{tg\cdots gg\cdots g}C_{g}(\mathcal {A}) \bigr]^{\frac{1}{2}} \bigr\} \\ &\geq \min_{i\in\bar{S},j\in S}\frac{1}{2} \bigl\{ r_{i}^{j}( \mathcal{A}) +\bigl[\bigl(r_{i}^{j}(\mathcal{A}) \bigr)^{2}+4a_{ij\cdots jj\cdots j}C_{j}(\mathcal {A}) \bigr]^{\frac{1}{2}} \bigr\} \geq \hat{L}^{\bar{S}}(\mathcal{A}). \end{aligned}$$

Assume that $\mathcal{A}$ is a nonnegative rectangular tensor, then by Lemma 3 of [2] and similar to the proof of Theorem 2 of [2], we can prove that the first inequality in (7) holds. The conclusion follows from what we have proved. □

Next, a comparison theorem for these bounds in Theorem 2 and Theorem 4 of [2] is given.

### Theorem 3

*Let* $\mathcal{A}=(a_{i_{1}\cdots i_{m}})\in\mathbb {R}^{[p,q;n,n]}_{+}$, *S* *be a nonempty proper subset of* *N*. *Then the bounds in Theorem* 2 *are better than those in Theorem* 4 *of* [2], *that is*,

$$\begin{aligned} \min_{1\leq i,j\leq n} \bigl\{ R_{i}(\mathcal{A}),C_{j}( \mathcal{A}) \bigr\} \leq L^{S}(\mathcal{A})\leq U^{S}( \mathcal{A})\leq\max_{1\leq i, j\leq n} \bigl\{ R_{i}( \mathcal{A}),C_{j}(\mathcal{A}) \bigr\} . \end{aligned}$$

### Proof

Here, only $L^{S}(\mathcal{A})=\min\{\hat{L}^{S}(\mathcal{A}),\hat{L}^{\bar {S}}(\mathcal{A}),\tilde{L}^{S}(\mathcal{A}),\tilde{L}^{\bar{S}}(\mathcal {A})\}\geq\min_{1\leq i,j\leq n} \{R_{i}(\mathcal{A}),C_{j}(\mathcal {A}) \}$ is proved. Similarly, we can also prove that $U^{S}(\mathcal{A})\leq\max_{1\leq i, j\leq n} \{R_{i}(\mathcal {A}),C_{j}(\mathcal{A}) \}$. Without loss of generality, assume that $L^{S}(\mathcal{A})=\hat{L}^{S}(\mathcal{A})$, that is, there are two indexes $i\in S,j\in\bar{S}$ such that

$$L^{S}(\mathcal{A})=\frac{1}{2} \bigl\{ r_{i}^{j}( \mathcal{A}) +\bigl[\bigl(r_{i}^{j}(\mathcal{A}) \bigr)^{2}+4a_{ij\cdots jj\cdots j}\min\bigl\{ R_{j}(\mathcal {A}),C_{j}(\mathcal{A})\bigr\} \bigr]^{\frac{1}{2}} \bigr\} $$

(we can prove it similarly if $L^{S}(\mathcal{A})=\hat{L}^{\bar {S}}(\mathcal{A}),\tilde{L}^{S}(\mathcal{A}),\tilde{L}^{\bar{S}}(\mathcal {A})$, respectively). Now, we divide the proof into two cases as follows.

Case I: Assume that

$$L^{S}(\mathcal{A})=\frac{1}{2} \bigl\{ r_{i}^{j}( \mathcal{A}) +\bigl[\bigl(r_{i}^{j}(\mathcal{A}) \bigr)^{2}+4a_{ij\cdots jj\cdots j}R_{j}(\mathcal {A}) \bigr]^{\frac{1}{2}} \bigr\} . $$

(i) If $R_{i}(\mathcal{A})\geq R_{j}(\mathcal{A})$, then $a_{ij\cdots jj\cdots j}\geq R_{j}(\mathcal{A})-r_{i}^{j}(\mathcal{A})$. When $R_{j}(\mathcal{A})-r_{i}^{j}(\mathcal{A})>0$, we have

$$\begin{aligned} L^{S}(\mathcal{A})&\geq \frac{1}{2} \bigl\{ r_{i}^{j}( \mathcal {A})+\bigl[\bigl(r_{i}^{j}(\mathcal{A}) \bigr)^{2}+4\bigl(R_{j}(\mathcal{A})-r_{i}^{j}( \mathcal {A})\bigr)R_{j}(\mathcal{A})\bigr]^{\frac{1}{2}} \bigr\} \\ &=\frac{1}{2} \bigl\{ r_{i}^{j}(\mathcal{A}) + \bigl[ \bigl(2R_{j}(\mathcal{A})-r_{i}^{j}(\mathcal{A}) \bigr)^{2} \bigr]^{\frac{1}{2}} \bigr\} \\ &= \frac{1}{2} \bigl\{ r_{i}^{j}(\mathcal{A}) +2R_{j}(\mathcal{A})-r_{i}^{j}(\mathcal{A}) \bigr\} \\ &= R_{j}(\mathcal{A}) \\ &\geq \min_{j\in\bar{S}}R_{j}(\mathcal{A}) \\ &\geq \min_{1\leq i, j\leq n} \bigl\{ R_{i}( \mathcal{A}),C_{j}(\mathcal {A}) \bigr\} . \end{aligned}$$

And when $R_{j}(\mathcal{A})-r_{i}^{j}(\mathcal{A})\leq0$, i.e., $r_{i}^{j}(\mathcal{A})\geq R_{j}(\mathcal{A})$, we have

$$\begin{aligned} L^{S}(\mathcal{A})&\geq \frac{1}{2} \bigl\{ r_{i}^{j}( \mathcal{A}) + \bigl[\bigl(r_{i}^{j}(\mathcal{A}) \bigr)^{2} \bigr]^{\frac{1}{2}} \bigr\} =r_{i}^{j}( \mathcal{A}) \geq R_{j}(\mathcal{A})\geq\min_{j\in\bar{S}}R_{j}( \mathcal{A}) \\ &\geq \min_{1\leq i, j\leq n} \bigl\{ R_{i}(\mathcal{A}),C_{j}( \mathcal {A}) \bigr\} . \end{aligned}$$

(ii) If $R_{i}(\mathcal{A})< R_{j}(\mathcal{A})$, then

$$\begin{aligned} L^{S}(\mathcal{A})&\geq \frac{1}{2} \bigl\{ r_{i}^{j}( \mathcal{A}) +\bigl[\bigl(r_{i}^{j}(\mathcal{A}) \bigr)^{2}+4a_{ij\cdots jj\cdots j}R_{i}(\mathcal {A}) \bigr]^{\frac{1}{2}} \bigr\} \\ &= \frac{1}{2} \bigl\{ r_{i}^{j}(\mathcal{A}) +\bigl[ \bigl(r_{i}^{j}(\mathcal{A})\bigr)^{2}+4a_{ij\cdots jj\cdots j} \bigl(r_{i}^{j}(\mathcal {A})+a_{ij\cdots jj\cdots j}\bigr) \bigr]^{\frac{1}{2}} \bigr\} \\ &= \frac{1}{2} \bigl\{ r_{i}^{j}(\mathcal{A}) +\bigl[ \bigl(r_{i}^{j}(\mathcal{A})+2a_{ij\cdots jj\cdots j} \bigr)^{2}\bigr]^{\frac{1}{2}} \bigr\} \\ &= r_{i}^{j}(\mathcal{A})+a_{ij\cdots jj\cdots j} \\ &= R_{i}(\mathcal{A}) \\ &\geq \min_{i\in S}R_{i}(\mathcal{A}) \\ &\geq \min_{1\leq i, j\leq n} \bigl\{ R_{i}( \mathcal{A}),C_{j}(\mathcal {A}) \bigr\} . \end{aligned}$$

Case II: Assume that

$$L^{S}(\mathcal{A})=\frac{1}{2} \bigl\{ r_{i}^{j}( \mathcal{A}) +\bigl[\bigl(r_{i}^{j}(\mathcal{A}) \bigr)^{2}+4a_{ij\cdots jj\cdots j}C_{j}(\mathcal {A}) \bigr]^{\frac{1}{2}} \bigr\} . $$

Similar to the proof of Case I, we have $L^{S}(\mathcal{A})\geq\min_{1\leq i, j\leq n} \{R_{i}(\mathcal {A}),C_{j}(\mathcal{A}) \}$. The conclusion follows from what we have proved. □

## Numerical examples

In the following, two numerical examples are given to verify the theoretical results.

### Example 1

Let $\mathcal{A}\in\mathbb{R}^{[2,2;3,3]}_{+}$ with entries defined as follows:

$$\begin{aligned} &\mathcal{A}(:,:,1,1)=\left [ \begin{matrix}0& 0& 0\\ 11& 0& 0\\ 0& 0& 0 \end{matrix} \right ], \qquad \mathcal{A}(:,:,2,1)=\left [ \begin{matrix} 0& 0& 0\\ 4& 6& 3\\ 10& 0& 3 \end{matrix} \right ], \\ &\mathcal{A}(:,:,3,1) \left [ \begin{matrix}0& 0& 0\\ 1& 1& 2\\ 7& 2& 2 \end{matrix} \right ], \qquad \mathcal{A}(:,:,1,2)=\left [ \begin{matrix}0& 0& 0\\ 0& 1& 0\\ 1& 0& 0 \end{matrix} \right ], \\ &\mathcal{A}(:,:,2,2)=\left [ \begin{matrix}0& 1& 0\\ 0& 2& 1\\ 0& 2& 3 \end{matrix} \right ], \qquad \mathcal{A}(:,:,3,2)=\left [ \begin{matrix}0& 0& 0\\ 2& 2& 2\\ 6& 2& 1 \end{matrix} \right ], \\ &\mathcal{A}(:,:,1,3)=\left [ \begin{matrix}0& 0& 0\\ 2& 1& 2\\ 0& 0& 0 \end{matrix} \right ], \qquad \mathcal{A}(:,:,2,3)=\left [ \begin{matrix}0& 0& 0\\ 2& 3& 1\\ 1& 1& 3 \end{matrix} \right ], \\ &\mathcal{A}(:,:,3,3)\left [ \begin{matrix} 2& 1& 1\\ 3& 2& 3\\ 2& 1& 1 \end{matrix} \right ]. \end{aligned}$$

By computation, we get that all different singular values of $\mathcal {A}$ are $-4.9395, -0.5833$, $-0.4341, -0.1977, 0, 0.0094, 0.0907, 1.0825, 1.2405, 1.5334, 1.8418, 2.3125, 5.8540, 6.1494$, $6.6525, 8.0225$ and 31.1680.

(i) An *S*-type singular value inclusion set.

Let $S=\{1\}$. Obviously, $\bar{S}=\{2,3\}$. By Theorem 1, the *S*-type singular inclusion set is

$$\begin{aligned} \Upsilon^{S}(\mathcal{A})=\bigl\{ z\in{\mathbb{C}}: \vert z \vert \leq49.9629\bigr\} . \end{aligned}$$

**Figure 1:**
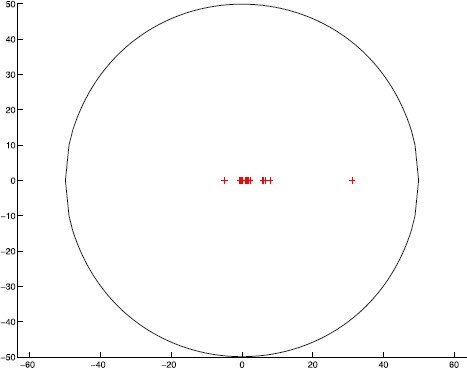
**The singular value inclusion set** $\pmb{\Upsilon ^{S}(\mathcal{A})}$ **and the exact singular values.**

The singular value inclusion set $\Upsilon^{S}(\mathcal{A})$ and the exact singular values are drawn in Figure 1, where $\Upsilon^{S}(\mathcal{A})$ is represented by black solid boundary and the exact singular values are plotted by red ‘+’. It is easy to see that $\Upsilon^{S}(\mathcal{A})$ can capture all singular values of $\mathcal{A}$ from Figure 1.

(ii) The bounds of the largest singular value.

By Theorem 4 of [2], we have

$$5\leq\lambda_{0}\leq57. $$

Let $S=\{1\}, \bar{S}=\{2,3\}$. By Theorem 2, we have

$$9.0711\leq\lambda_{0}\leq49.9629. $$

In fact, $\lambda_{0}=31.1680$. This example shows that the bounds in Theorem 2 are better than those in Theorem 4 of [2].

### Example 2

Let $\mathcal{A}\in\mathbb{R}^{[2,2;2,2]}_{+}$ with entries defined as follows:

$$a_{1111}=a_{1112}=a_{1222}=a_{2112}=a_{2121}=a_{2221}=1, $$

other $a_{ijkl}=0$. By computation, we get that all different singular values of $\mathcal {A}$ are $0, 0.8226, 1, 3$.

(i) An *S*-type singular value inclusion set.

Let $S=\{1\}$. Obviously, $\bar{S}=\{2,3\}$. By Theorem 1, the *S*-type singular inclusion set is

$$\begin{aligned} \Upsilon^{S}(\mathcal{A})=\bigl\{ z\in{\mathbb{C}}: \vert z \vert \leq3\bigr\} . \end{aligned}$$

**Figure 2:**
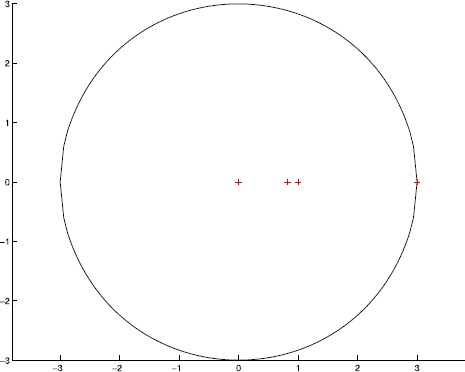
**The singular value inclusion set** $\pmb{\Upsilon ^{S}(\mathcal{A})}$ **and the exact singular values.**

The singular value inclusion set $\Upsilon^{S}(\mathcal{A})$ and the exact singular values are drawn in Figure 2, where $\Upsilon^{S}(\mathcal{A})$ is represented by black solid boundary and the exact singular values are plotted by red ‘+’. It is easy to see that $\Upsilon^{S}(\mathcal{A})$ captures exactly all singular values of $\mathcal{A}$ from Figure 2.

(ii) The bounds of the largest singular value.

By Theorem 2, we have

$$3\leq\lambda_{0}\leq3. $$

In fact, $\lambda_{0}=3$. This example shows that the bounds in Theorem 2 are sharp.

## Conclusions

In this paper, we give an *S*-type singular value inclusion set $\Upsilon^{S}(\mathcal{A})$ for rectangular tensors. As an application of this set, an *S*-type upper bound $U^{S}(\mathcal {A})$ and an *S*-type lower bound $L^{S}(\mathcal{A})$ for the largest singular value $\lambda_{0}$ of a nonnegative rectangular tensor $\mathcal {A}$ are obtained and proved to be sharper than those in [2]. Then, an interesting problem is how to pick *S* to make $\Upsilon ^{S}(\mathcal{A})$ as tight as possible. But it is difficult when the dimension of the tensor $\mathcal{A}$ is large. We will continue to study this problem in the future.
